# Feasibility study of mindfulness-based cognitive therapy for anxiety disorders in a Japanese setting

**DOI:** 10.1186/s13104-018-3744-4

**Published:** 2018-09-06

**Authors:** Mitsuhiro Sado, Sunre Park, Akira Ninomiya, Yasuko Sato, Daisuke Fujisawa, Joichiro Shirahase, Masaru Mimura

**Affiliations:** 1 Department of Neuropsychiatry, Keio University School of Medicine, Tokyo, Japan; 2 Center for Stress Research, Keio University, Tokyo, Japan; 3 Faculty of Nursing and Medicine Care, Keio University, Tokyo, Japan; 4 Palliative Care Center, Keio University Hospital, Tokyo, Japan; 5 Department of Nursing, National Hospital Organization Tokyo Medical Center, Tokyo, Japan; 6 Division of Patient Safety, Department of Neuropsychiatry, Keio University School of Medicine, Tokyo, Japan

**Keywords:** Mindfulness, Anxiety disorders, Mindfulness-based cognitive behavioral therapy

## Abstract

**Objective:**

Mindfulness-based cognitive therapy (MBCT) could be a treatment option for anxiety disorders. Although its effectiveness under conditions of low pharmacotherapy rates has been demonstrated, its effectiveness under condition of high pharmacotherapy rate is still unknown. The aim of the study was to evaluate effectiveness of MBCT under the context of high pharmacotherapy rates.

**Results:**

A single arm with pre-post comparison design was adopted. Those who had any diagnosis of anxiety disorders, between the ages of 20 and 74, were included. Participants attended 8 weekly 2-hour-long sessions followed by 2 monthly boosters. Evaluation was conducted at baseline, in the middle, at end of the intervention, and at follow-up. The State-Trait Anxiety Inventory (STAI)-state was set as the primary outcome. Pre-post analyses with mixed-effect models repeated measures were conducted. Fourteen patients were involved. The mean age was 45.0, and 71.4% were female. The mean change in the STAI-state at every point showed statistically significant improvement. The STAI-trait also showed improvement at a high significance level from the very early stages. The participants showed significant improvement at least one point in some other secondary outcomes.

*Trial registration* Retrospectively registered at the University Hospital Medical Information Network on 1st August 2013 (ID: UMIN000011347)

## Introduction

Anxiety disorders are among the most prevalent and long-term mental disorders worldwide. The prevalence rates are estimated to be 18.1% in the US [1], 6.4% in Europe [2], and 5.5% in Japan [3]. The accumulative remission rates within 8 years for social anxiety disorders, panic disorders with agoraphobia, and generalized anxiety disorders remain low, at: 31, 38, and 49%, respectively [4]. The intractable feature of the condition is one of its aspects that boost prevalence.

Such high prevalence considerably burdens the society. The latest disability studies [5, 6] revealed that anxiety disorders represent the 6th leading disease in terms of years of life lived with a disability in 2010. The burden converted into monetary cost manifests the magnitude. The societal cost of anxiety disorders rose to USD 42.3 billion in the US in 1990 [7], GBP 8.9 billion in England in 2007 [8], and JPY 2.4 trillion in Japan in 2008 [9].

Although pharmacotherapy and individualized cognitive behavior therapy (CBT) were recommended as the 1st line treatment among the clinical guidelines [10–12], more patients chose CBT if it was available. However, psychotherapists’ scarcity restricts the dissemination of adequate individualized CBT. Therefore, the development of another form of psychotherapy, which is as cost effective and effective as CBT, is necessary.

Mindfulness-based cognitive therapy (MBCT) would meet these requirements. It is a form of group psychotherapy combining the essence of CBT and mindfulness-based stress reduction (MBSR) program, which was developed and brought into the healthcare arena by Jon Kabat-Zinn in the 1970s [13]. MBCT aims to cultivate mindfulness and intentional non-judgmental awareness in present-moment experiences [14]. This therapy could be more cost effective than individualized CBT because MBCT is normally provided in a group setting.

The effectiveness of MBCT has been demonstrated in various treatment areas: relapse prevention of depressive episodes [15–18], psychological distress among cancer patients [19–22], chronic pain [23], and so on [24]. Even in the area of anxiety disorder treatment, several studies have already reported significant favorable effects [25–29]. However, because pharmacotherapy rates among the participants in these studies are considerably low (ranging from 0 to 39%), its effectiveness under settings in which the vast majority of patients have already experienced pharmacotherapy (e.g., in Japan the pharmacotherapy rate among patients with anxiety disorders is 86% [30]) is still unknown. Therefore, we aimed to investigate the feasibility and effectiveness of MBCT as a pilot study with a single arm for patients with anxiety disorders.

## Main text

### Method

#### Design

A single arm study was used to test MBCT’s feasibility and effectiveness with a pre-post comparison. The study was conducted between March 2013 and September 2014.

#### Ethics approval and consent to participate

This study was approved by the ethical committee at Keio University School of Medicine and registered at the University Hospital Medical Information Network on 1st August 2013 (ID: UMIN000011347).

#### Participants

Inclusion criteria for this study were: (1) any diagnosis of panic disorder, social anxiety disorder, obsessive compulsive disorder, or generalized anxiety disorder using the Diagnostic and Statistical Manual of Mental Disorders 4th edition; (2) aged between 20 and 74 years; and (3) able to provide consent in writing. The exclusion criteria were: (1) any past history of substance-related disorders/manic or psychotic episode/receiving mindfulness-based intervention, (2) impairment in cognitive function, (3) antisocial personality disorder, (4) severe suicidal ideation, and (5) expected difficulty in following up 4 months after the start of the intervention.

#### Procedure

The participants were recruited from the Department of Neuropsychiatry, Keio University Hospital. After providing written consent, they attended an 8-week course of MBCT for anxiety, followed by 2 monthly booster sessions. Evaluation was conducted before the intervention (0 weeks) (T0), during it (4 weeks) (T1), at its end (8 weeks) (T2), and during the follow-up periods (1 month (T3) and 2 months after the completion of the program (T4)).

#### Intervention

Because the original MBCT [13] targets relapse prevention for depression, we made minimal modifications to the original program in order to ensure that it fits with anxiety disorders. Specifically, we revised the psychoeducational part in Session 4 so that it would be relevant to anxiety disorders. The details of the program are shown in Table 1.

**Table 1:** Contents of the program

Session	Theme	Contents
1	Automatic pilot	Psychoeducation: what is mindfulness
		Exercise: mindfulness eating (“Raisin exercise”)/body scan
		Homework: mindfulness of a routine activity/body scan
2	Dealing with barriers	Psychoeducation: association of mood and thoughts
		Exercise: thoughts and feelings exercise/body scan/mindful breathing meditation
		Homework: body scan/breathing meditation/pleasant events calendar
3	Mindfulness of the breath	Psychoeducation: awareness of mind wandering and focuing on the breath
		Exercise: breathing meditation/gentle yoga/mindful walking
		Homework: breathing meditation/gentle yoga/mindful walking/unpleasant events calendar
4	Staying present	Psychoeducation: staying present/about anxiety symptoms^a^
		Exercise: meditation of sounds and thoughts/breathing meditation
		Homework: meditation of sounds and thoughts/breathing meditation/3-min breathing space
5	Allowing/letting be	Psychoeducation: exploring difficulty
		Exercise: breathing meditation/meditation of sounds and thoughts/exploring difficulty
		Homework: breathing meditation/meditation of sounds and thoughts/exploring difficulty/3-min breathing space
6	Thoughts are not facts	Psychoeducation: cognitive biases
		Exercise: breathing meditation/meditation of sounds and thoughts/exploring difficulty
		Homework: breathing meditation/meditation of sounds and thoughts/exploring difficulty/3-min breathing space
7	How can I best take care of myself?	Psychoeducation: choosing functional behaviors/behavioral activation/identifying triggers
		Exercise: mindfulness meditation of sounds and thoughts/breathing meditation
		Homework: meditation of sounds and thoughts/breathing meditation/3-min breathing space + action plan
8	Using what has been learned to deal with future mood	Personal reflections of course/plans for future practice and strategies for maintaining momentum/farewell
		Exercise: body scan/breathing meditation

^a^ The lecture relevant to depression was replaced by that about anxiety in session 4

This program consists of 8 weekly 2-hour-long sessions. Each session consists of three parts: (1) practicing meditation and yoga, (2) sharing experiences, and (3) psychoeducational portions. The participants were encouraged to practice daily for 30 min to 1 h at home with audio CD instructions and perform other exercises such as monitoring positive or negative feelings.

The program was led by a psychiatrist (MS) and a nurse/clinical psychologist (SP), both of whom had practiced mindful meditation for more than 2 years with experience in attending MBSR and MBCT retreats.

#### Outcomes

We used the following scales to measure outcomes, all of which had already been validated in Japanese.

##### Primary

We set the State-Trait Anxiety Inventory (STAI)-state as the primary outcome. The STAI is a commonly used measure of state and trait anxiety [31].

##### Secondary

We also used the following scales for secondary outcomes: the STAI-trait, 6-item Kessler Psychological Distress Scale (K-6), Center for Epidemilogic Studies Depression Scale (CES-D), EuroQol 5 Dimension (EQ-5D), Five Facet Mindfulness Questionnaire (FFMQ), 36-Item Short-Form Health Survey (SF-36), Mobility Inventory for Agoraphobia (MIA), and Liebowitz Social Anxiety Scale (LSAS). The details of each scale are as follows.

*K*-*6* The K6 scale was designed to be sensitive to the threshold for the clinically significant range of the distribution of nonspecific distress in an effort to maximize its ability to discriminate cases of serious mental illness from non-cases [32].

*CES*-*D* The CES-D is a short self-report scale designed to measure depressive symptomatology among the general population [33]. The scale contains 20 items that ask how often over the past week the patients experienced symptoms associated with depression.

*EQ*-*5D* EQ-5D is a standardized instrument for use as a measure of health outcomes [34]. It provides a simple descriptive profile and a single index value for health status.

*FFMQ* The FFMQ is based on a factor analytic study of five independently developed mindfulness questionnaires [35]. The five facets are observing, describing, acting with awareness, the non-judging of inner experience, and non-reactivity to inner experience.

*SF*-*36* The SF-36 is a set of generic, coherent, and easily administered quality-of-life measures. These measures rely on patient self-report [36]. It consists of eight sections: vitality, physical functioning, bodily pain, general health perceptions, physical role functioning, emotional role functioning, social role functioning, and mental health [37, 38].

*MIA* The MIA is a measurement of self-reported agoraphobic avoidance behavior and frequency of panic attacks [39]. Respondents rate 26 items using Likert-type scales ranging from 1 (never avoid) to 5 (always avoid) to indicate how much they avoid various situations due to anxiety or discomfort when they are accompanied by a trusted companion and when they are alone.

*LSAS* The LSAS is an instrument used to assess the range of social interactions and performance situations that a patient fears; it assists in the diagnosis of social anxiety disorder [32]. The scale features 24 items, which are divided into two subscales. Of these, 13 questions relate to performance anxiety and 11 to social situations.

#### Analyses

Changes in mean scores between the baseline and each observational period for each scale were tested with mixed-effect models, repeated-measures, and intention-to-treat analyses. Age, sex, and intervention were imputed into the model as coefficients. A 5% significance level was adopted for all statistical analyses. STATA ver.15 was used to conduct the statistical analysis.

### Results

#### Basic participant characteristics

Fourteen patients were involved in the research. As shown in Table 2, mean age (standard deviation: sd) was 45.0 (14.1), and 71.4% were female. With respect to primary diagnosis, six participants had panic disorders, while five and three had social anxiety disorder and obsessive compulsive disorder, respectively. Average treatment duration at the start of the intervention was 13.4 years (7.4) and the rate of pharmacotherapy use was 93% (71.4% and 50.0% used antidepressants and benzodiazepine, respectively). All but one of the participants completed the program and the average number of program attendance was 7.4 (1.1). The reason for dropout was the deterioration of respiratory symptoms that were originally comorbid.

**Table 2:** Baseline characteristics of the participants

Characteristics	Mean/n	sd/%
Age	45.0	14.1
Gender (female)	10	71.4
Diagnosis		
Panic disorder	6	42.9
SAD	5	35.7
OCD	3	21.4
Duration from onset (years)	13.4	7.4
Medication	13	92.9
Antidepressants	10	71.4
Benzodiazepine	7	50.0
Mood stabilizer	2	14.3
Antipsychotics	2	14.3
Frequency of attendance	7.4	1.1

*SAD* social anxiety disorder, *OCD* obsessive compulsive disorder

#### Primary outcome

As described in Table 3, the mean change (sd) from baseline in STAI-state at every point showed statistically significant improvements: − 6.14 (2.70) at T1, − 11.66 (2.78) at T2, − 8.12 (2.78) at T3, and − 6.58 (2.78) at T4.

**Table 3:** Outcomes scores at each assessment point with comparison to baseline

Mean score at baseline and mean differences compared to baseline											
	n	Baseline		4 week		8 week		12 week		16 week	
		Mean	se	Mean difference	se	Mean difference	se	Mean difference	se	Mean difference	se
STAI-state	14	52.6	2.8	− 6.14	2.70	− 11.66	2.78	− 8.12	2.78	− 6.58	2.78
*P* value				*0.02*		*< 0.001*		*< 0.01*		*0.02*	
STAI-trait	14	55.9	3.0	− 3.86	1.85	− 6.55	1.90	− 4.86	1.90	− 3.93	1.90
*P* value				*0.04*		*< 0.01*		*0.01*		*0.04*	
K-6	14	8.6	1.6	− 1.07	1.17	− 2.68	1.20	− 1.68	1.20	0.16	1.20
*P* value				0.36		*0.03*		0.16		0.89	
CESD	14	17.7	3.0	− 0.71	2.03	− 1.46	2.09	− 3.85	2.09	1.69	2.09
*P* value				0.73		0.48		0.07		0.42	
SF36 PCS	14	54.1	2.8	− 0.57	2.02	1.68	2.07	0.52	2.07	1.32	2.07
*P* value				0.78		0.42		0.80		0.53	
SF36MCS	14	41.4	3.6	− 2.17	2.18	2.11	2.25	2.27	2.25	− 1.33	2.25
*P* value				0.32		0.35		0.31		0.55	
SF36 RCS	14	42.5	4.3	3.13	2.84	3.03	2.92	4.50	2.92	2.29	2.92
*P* value				0.27		0.30		0.12		0.43	
FFMQ (total)	14	110.4	4.9	− 1.42	2.25	2.51	2.31	0.96	2.31	− 1.17	2.31
*P* value				0.53		0.28		0.68		0.61	
FFMQ (observe)	14	22.6	1.5	− 0.93	0.99	0.07	1.01	0.14	1.01	0.77	1.01
*P* value				0.35		0.94		0.89		0.45	
FFMQ (noreact)	14	16.6	1.7	− 0.79	0.84	0.93	0.87	0.81	0.87	0.39	0.87
*P* value				0.35		0.28		0.35		0.65	
FFMQ (nonjudgement)	14	23.9	2.0	2.00	1.17	2.62	1.21	2.54	1.21	0.77	1.21
*P* value				0.09		*0.03*		*0.04*		0.52	
FFMQ (descrive)	14	22.9	1.5	− 1.18	0.84	0.57	0.87	− 0.96	0.87	− 1.04	0.87
*P* value				0.16		0.51		0.27		0.23	
FFMQ (awareness)	14	24.5	1.6	− 0.52	1.11	− 1.69	1.15	− 1.58	1.15	− 2.07	1.15
*P* value				0.64		0.14		0.17		0.07	
EQ-5D	14	0.79	0.04	0.01	0.03	0.02	0.03	0.03	0.03	0.04	0.03
*P* value				0.79		0.41		0.22		0.09	
MIA (AAC)	6	1.88	0.29	− 0.08	0.09	− 0.18	0.09	− 0.13	0.09	− 0.24	0.09
*P* value				0.39		0.05		0.14		*< 0.01*	
MIA (AAL)	6	2.24	0.49	− 0.02	0.14	− 0.15	0.14	− 0.05	0.14	− 0.25	0.14
*P* value				0.87		0.28		0.71		0.07	
MIA (panic attack 1 weeks)	6	2.83	2.44	− 0.17	1.19	− 1.67	1.19	− 2.17	1.19	− 1.67	1.19
*P* value				0.89		0.16		0.07		0.16	
MIA (panic attack 3 weeks)	6	5.83	4.85	− 1.00	1.72	− 3.33	1.72	− 2.83	1.72	− 2.50	1.72
*P* value				0.56		0.05		0.10		0.15	
LSAS	5	73.40	13.41	− 12.00	6.81	− 3.63	7.42	− 7.38	7.42	− 9.13	7.42
*P* value				0.08		0.63		0.32		0.22	

#### Secondary outcome

As shown in Table 3, significant improvements were observed in multifarious scales. For STAI-trait, improvement was observed from the very early stages with high significance levels and continued until 2 months after the completion of the intervention. With respect to K-6, the participants showed significant improvements post-treatment, but these disappeared during follow-up. Although the total score did not show any change, significant improvements were noted on an FFMQ subscale (i.e., non-judgment) immediately and also at 1 month post-treatment. Moreover, the trend-level improvement in another subscale (i.e., awareness) at 2 months post-treatment was acknowledged.

With regard to the disease-specific scales, the MIA (AAC) score indicated significant improvements at 2 month follow-up, while only improvement trend was detected in MIA (AAL) at the end of the follow-up. No improvement was observed in LSAS, specifically, among the participants with social anxiety disorders.

### Discussion

To the best of our knowledge, this is the first study in Japan to evaluate the applicability and feasibility of the MBCT for anxiety disorders. The present results indicate that it would favorably affect the symptoms of anxiety disorders. The quite low attrition rate also demonstrated its feasibility even in settings under which pharmacotherapy had already been provided to the vast majority of participants.

The improvement in STAI-state with a high significance level that was observed even post-treatment indicates that the efficacy of MBCT would be sustained, even after the intervention. Another surprising result was that STAI-trait similarly improved significantly. This means the MBCT has the potential to remedy the trait that would affect the onset and continuity of anxiety symptoms from the very early stages. This might represent the peculiarity of mindfulness, which aims to transform the attitude toward unpleasant events rather than to remove the unpleasant experiences themselves.

We covered only two disease-specific scales: agoraphobia and panic attack (MIA) and social phobia (LSAS) due to the constraints of scale availability. The fact that MIA (AAC) showed significant immediate and 2-month-post-treatment improvements indicates that MBCT has the potential to be effective even for disease-specific symptoms.

Attention should be paid to the interpretation of the results of the FFMQ. The FFMQ is composed of five factors, and is supposed to converge at two higher factors: “self-regulated attention” and “orientation to experience” [40]. Although a previous study showed significant changes in all FFMQ factors pre- and post-intervention in the MBCT group [41], no changes were detected in this study except for “non-judging.” As indicated in previous studies, although numerous validation studies have been conducted to measure mindfulness, the results appear to vary [42]. The discrepancy between the previous and current studies possibly represents the nature of this scale. In addition, the discrepancy could be due to the small sample size of the current study. No, or quite limited, specific measures for anxiety in the CES-D, EQ-5D, and SF-36 would be a reason why no significant changes were observed in scores on these scales.

## Limitations

This study has some limitations. The sample size was too small to conduct subgroup analyses between different types of anxiety disorders. This also affected the generalizability of the results. The other limitation was that the study was performed with a single arm pre-post design. Therefore, it is necessary to conduct randomized controlled studies to accurately evaluate its effectiveness in future research.

## Abbreviations

AAC: Avoidance Accompanied Scale
AAL: Avoidance Alone Scale
CBT: cognitive behavior therapy
CES-D: Center for Epidemiologic Studies Depression Scale
EQ-5D: EuroQol 5 Dimension
FFMQ: Five Facet Mindfulness Questionnaire
K-6: the 6-item Kessler Psychological Distress Scale
LSAS: the Liebowitz Social Anxiety Scale
MBCT: mindfulness-based cognitive therapy
MBSR: mindfulness-based stress reduction
MIA: the Mobility Inventory for Agoraphobia
sd: standard deviation
SF-36: the 36-Item Short-Form Health Survey
STAI: the State-Trait Anxiety Inventory
